# Study on Allometric Growth and Digestive System Development in Larvae of Largemouth Bronze Gudgeon (*Coreius guichenoti*)

**DOI:** 10.3390/ani16121911

**Published:** 2026-06-19

**Authors:** Yu Zhao, Huan-Tao Qu, Jian Zhu, Yang Li, Ting-Ting Shu, Chao Cheng, Pei Chen

**Affiliations:** 1 Chinese Sturgeon Research Institute, China Three Gorges Corporation, Yichang 443100, China; 2 Hubei Key Laboratory of Three Gorges Project for Conservation of Fishes, Yichang 443100, China

**Keywords:** *Coreius guichenoti*, allometric growth, digestive system development, digestive enzyme activity, larval rearing

## Abstract

Understanding the early life history of the endangered largemouth bronze gudgeon (*C. guichenoti*) is crucial for optimizing artificial rearing and conservation strategies. To provide a unified scientific basis for these efforts, this study characterized the ontogeny of *C. guichenoti* larvae from hatching to 30 days post-hatch (dph) by integrating analyses of allometric growth, digestive histology, and enzyme activities. Morphometric analysis revealed that total length increased exponentially with age. Positive allometric growth was observed in swimming organs (pectoral, dorsal, and caudal fins) and eye diameter, indicating a priority in developing locomotion and sensory capabilities for survival in lotic environments. Histological examination showed that the digestive tract became patent by 4 dph, and functional structures were established by 5 dph, coinciding with yolk sac absorption and the initiation of exogenous feeding. The digestive system reached near-adult maturity by 20 dph. Enzyme activity profiles showed that trypsin activity increased continuously, peaking at 30 dph, while amylase and alkaline phosphatase peaked at 20 dph. Lipase activity remained low until a slight increase at 30 dph. These results indicate that *C. guichenoti* larvae adopt a “locomotion-first” strategy, with the digestive system becoming functional at 5 dph and maturing by 20 dph. These findings collectively define the critical windows for artificial propagation, specifically guiding the timing of initial feeding and feed formulation.

## Simple Summary

Largemouth bronze gudgeon (*Coreius guichenoti*) is an important fish species native to the Yangtze River that is currently facing population decline. To help protect this species, we need to understand how it grows in the wild. This study carefully observed the development of larvae from hatching to 30 days old. We found that the larvae prioritize developing their swimming fins and eyes very early to survive in strong currents, while their digestive system matures later to match the timing of when they start eating external food. We identified the exact days when they start eating and when their digestion becomes strong. By understanding these growth stages, we can provide clear guidance for breeding this fish in hatcheries, which will help save this endangered species.

## 1. Introduction

The early life history of fish represents a critical bottleneck determining recruitment success and population sustainability, particularly for species inhabiting dynamic riverine ecosystems [1,2,3]. During this sensitive period, larvae undergo a series of profound physiological and morphological transformations. They must transition from relying on endogenous yolk reserves to capturing and digesting exogenous food while simultaneously developing the locomotor apparatus necessary for survival in their specific habitat [4,5]. This complex process involves the coordinated development of the digestive system and external morphology, governed by both genetic programming and environmental cues.

The ontogeny of the digestive system in fish larvae is a stepwise process that begins with the formation of a simple digestive tube and progresses to a functionally mature organ capable of enzymatic hydrolysis and nutrient absorption [2,6]. In many cyprinids, including those endemic to the Yangtze River, the liver and pancreas often fuse into a diffuse hepatopancreatic complex during development, which plays a vital role in energy storage and metabolic regulation [7,8]. For stomachless species, the differentiation of the esophagus and intestine, along with the timely secretion of digestive enzymes such as trypsin and amylase, is particularly crucial for the successful initiation of feeding [9,10]. Understanding the histological and enzymatic timelines of this development is essential for identifying the “critical period” of larval vulnerability.

Concomitant with internal development, external morphological changes, particularly allometric growth, reflect adaptive responses to ecological pressures [2,4,11]. Allometric growth describes the differential growth rates of body parts relative to the whole body. In aquaculture and ecology, analyzing allometry is vital because it reveals how energy is allocated to functionally important organs—such as fins for locomotion or eyes for predation—often at the expense of other body regions [12,13]. This prioritization is a key strategy for surviving in challenging environments, such as swift-flowing rivers where maintaining position is energetically costly [14,15].

The largemouth bronze gudgeon (*C. guichenoti*) is an ecologically significant cyprinid endemic to the upper Yangtze River basin [16]. Historically abundant, its populations have declined precipitously due to habitat alteration caused by hydropower dams and overfishing, necessitating urgent conservation and artificial propagation efforts [16,17]. While previous studies have documented its embryonic development and adult feeding habits, a comprehensive, multi-system analysis integrating allometric growth, digestive histology, and enzyme activity profiles during the early larval stage is currently lacking [18,19,20].

Therefore, this study integrates analyses of allometric growth, digestive histology, and enzyme activity profiles from hatching to 30 days post-hatch. The primary objective is to elucidate the adaptive “locomotion-first” strategy of *C. guichenoti* larvae and define the critical physiological windows for feeding, thereby providing a consolidated scientific basis for optimizing artificial propagation protocols and supporting the conservation of this endangered species.

## 2. Materials and Methods

### 2.1. Broodstock and Artificial Spawning

Broodstock of *C. guichenoti* were wild-caught from the Jinsha River, with an age range of 6–9 years. The fish were acclimated to artificial culture conditions for more than one year before spawning induction. In the artificial spawning procedure, 5 females (mean weight: 968 ± 24.3 g) and 9 males (mean weight: 792 ± 18.3 g) were induced. The spawning induction method involved two injections for females and one injection for males, with the second injection for females administered simultaneously with the injection for males. All injections were given at the base of the pectoral fin. The hormones used were LHRH-A (Luteinizing Hormone-Releasing Hormone Agonist; Ningbo Second Hormone Factory, Ningbo, China) and DOM (Dopamine Antagonist; Ningbo Second Hormone Factory, Ningbo, China). For females, the first injection consisted of LHRH-A at 8 μg/kg body weight and DOM at 8 μg/kg body weight. After a 12 h interval, a second injection was given to females at LHRH-A 12 μg/kg body weight and DOM 12 μg/kg body weight. Males received a single injection of LHRH-A 6 μg/kg body weight and DOM 6 μg/kg body weight. The latency period after hormone administration was 10–16 h. Artificial fertilization was performed within this latency period. The eggs from each female were mixed with sperm from at least three males. After combining eggs and sperm, water was added and the mixture was gently swirled for more than one minute. Fertilized eggs were then transferred to incubation facilities.

### 2.2. Incubation and Larval Rearing

Fertilized eggs were incubated in conical hatching jars with an upward water flow to simulate natural lotic conditions. Hatching began at 3 days post-fertilization (dpf). Incubation and larval rearing were conducted in open-flow systems supplied with treated river water (sedimentation, filtration, aeration, and oxygenation). Larvae were reared in circular fiberglass tanks (1 m diameter) at a density of 1800 ind/m^3^. A total of 3 tanks were used as replicates. The flow velocity was kept below 0.03 m/s. Water quality parameters were maintained as follows: temperature 21–24 °C, dissolved oxygen 6.5–7.5 mg/L, ammonia nitrogen ≤ 0.1 mg/L, and nitrite ≤ 0.05 mg/L. The photoperiod was set at 12 h light: 12 h dark. The larval survival rate during the experimental period was >95%.

### 2.3. Feeding Protocol

At the onset of exogenous feeding (approximately 5 days post-hatch, dph), larvae were fed zooplankton cultured in outdoor ponds. The species used were: rotifer *Keratella cochlearis* (crude protein: 59.4 ± 3.1%, crude lipid: 8.1 ± 1.3%), cladoceran *Bosmina longirostris* (crude protein: 57.8 ± 4.5% protein, crude lipid: 9.2 ± 2.0%), and copepod *Mesocyclops leuckarti* (crude protein: 60.3 ± 4.1%, crude lipid: 16.3 ± 1.6%). Zooplankton were cultured according to the following method. Outdoor ponds were disinfected and sun-dried, then organic fertilizer made from livestock manure was applied at 0.5 kg per square meter. River water was then added to a depth of 0.7–1 m. After water addition, no further water was supplied, and the ponds were kept in a closed state. EM bacterial solution (containing lactic acid bacteria, yeast, and photosynthetic bacteria; total bacterial count ≥ 1.5 × 10^8^ CFU/mL) was sprayed at 80 mL per square meter. After 10–14 days, the ponds became rich with natural zooplankton, which were then harvested and fed to the larvae.

Feeding schedule: from 5 to 8 dph, rotifers were provided at 2–5 ind/mL; from 8 to 15 dph, juvenile cladocerans were added at 1–2 ind/mL alongside rotifers; after 15 dph, the diet consisted solely of cladocerans (3–5 ind/mL) and copepods (0.5–1 ind/mL). Zooplankton were offered to satiation three times daily (08:00, 14:00, and 20:00). Tanks were siphoned daily to remove debris and dead larvae prior to the first feeding.

### 2.4. Sampling Procedure

Sampling commenced on the day of hatching (0 dph). During the experiment, samples were collected at the same time of day before the morning feeding. For morphological measurements: larvae were collected daily from 0 to 10 dph, and every other day from 10 to 30 dph. At each time point, twelve individuals were randomly selected. For histological observation: larvae were collected daily from 0 to 30 dph. At each time point, twelve individuals were fixed. For enzyme activity assays: larvae were collected daily from 0 to 10 dph, every 2 days from 11 to 19 dph, and every 5 days from 20 to 30 dph. At each time point, nine samples were prepared. The number of larvae per sample varied by developmental stage: from 0 to 10 dph, each sample consisted of six larvae; from 10 to 20 dph, each sample consisted of three larvae; from 20 to 30 dph, each sample consisted of a single larva. Samples were taken evenly from three replicate tanks to ensure representativeness.

### 2.5. Morphological Measurements

**Figure 1:**
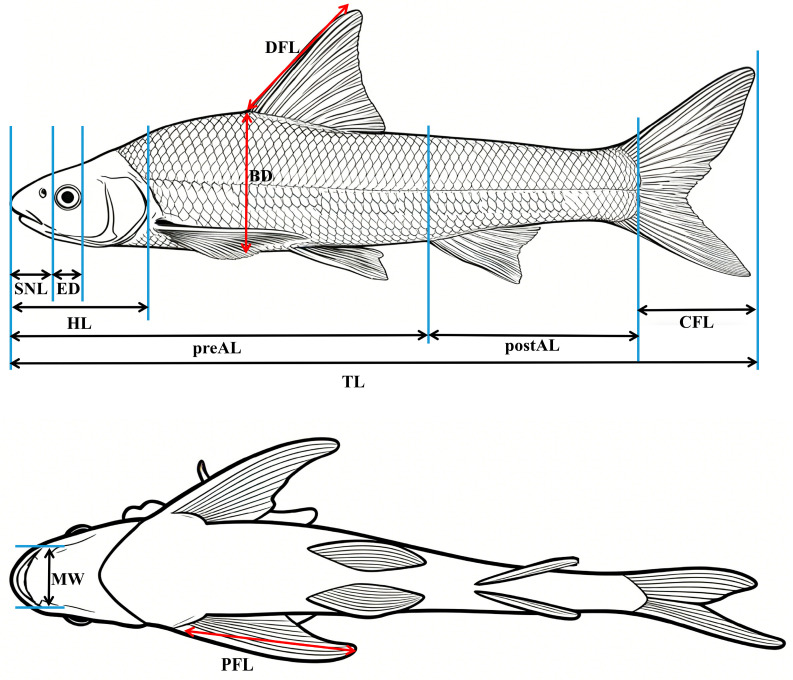
Schematic diagram of morphological and metric indicators in the larvae. TL: total length; HL: head length; ED: eye diameter; SNL: snout length; MW: mouth width; PFL: pectoral fin length; DFL: dorsal fin length; CFL: caudal fin length; preAL: pre-anal length; postAL: post-anal length; BD: body depth.

Larvae were anesthetized with MS-222, gently blotted dry, and photographed under a stereomicroscope (SZX10, Olympus, Tokyo, Japan). A total of 11 morphological traits were measured using Lightools software (version 4.10.17659.20200906; ToupTek, Hangzhou, China) following established methods [21,22,23]: total length, head length, eye diameter, rostrum length, mouth width, pectoral fin length, dorsal fin length, caudal fin length, pre-anal length, post-anal length, and body depth (Figure 1). Caudal fin length was measured from 0 dph, whereas the lengths of other fins were recorded only after their morphological differentiation was complete. For all fins, length was defined as the straight-line distance from the origin of the outer fin base to the distal tip of the longest fin ray.

### 2.6. Histological Observation

Larvae were fixed in 4% paraformaldehyde (Biosharp, Hefei, China) for 48 h at room temperature. After fixation, samples were dehydrated through a graded ethanol (Biosharp, Hefei, China) series (50%, 70%, 80%, 95%, and 100% ethanol), cleared in xylene (Xilong Scientific, Shantou, China), and embedded in paraffin wax (Xilong Scientific, Shantou, China). Serial sections were cut at 10 μm thickness using a rotary microtome (HS3315, ZEEDO, Jinhua, China). Sections were then dewaxed in xylene, rehydrated through a descending ethanol series, and stained with hematoxylin and eosin (H.E., Baso, Zhuhai, China) following standard protocols [10,24]. Stained sections were mounted with neutral balsam (Solarbio, Beijing, China). Histological examination was performed under a light microscope (BX53, Olympus, Tokyo, Japan), and images were captured using a digital camera (E3ISPM, ToupTek, Hangzhou, China).

### 2.7. Digestive Enzyme Activities

#### 2.7.1. Sample Preparation

Samples were homogenized on ice in deionized water (1:9 *w*/*v* ratio) and centrifuged at 5000 r/min for 20 min at 4 °C. The supernatant was collected and used for subsequent enzyme activity and soluble protein assays. Whole larvae were used for samples collected before 15 dph, and dissected visceral masses were used from 15 dph onward.

#### 2.7.2. Biochemical Assays

Total soluble protein concentration was determined using the Coomassie Brilliant Blue method (total protein quantitation kit, Cat. No. A045-2, Nanjing Jiancheng Bioengineering Institute, Nanjing, China). Amylase activity was measured using a commercial kit (Cat. No. C016-1-1, starch-iodine colorimetric method) at 37 °C and with absorbance measured at 660 nm. Trypsin activity was measured using a commercial kit (Cat. No. A080-2-2, ultraviolet colorimetric method) at 37 °C and with absorbance measured at 253 nm. Lipase activity was measured using a commercial kit (Cat. No. A054-1-1, turbidimetric method) at 37 °C and with absorbance measured at 420 nm. Alkaline phosphatase activity was measured using a commercial kit (Cat. No. A059-1-1, visible light colorimetric method) at 37 °C and with absorbance measured at 520 nm. All measurements were performed following the manufacturer’s instructions. Enzyme activities were expressed as specific activity (U/mg prot) after normalization to soluble protein concentration.

### 2.8. Data Analysis

Allometric growth models were fitted using the minpack.lm package in Rstudio (version 2023.06.2-561; Posit Software, PBC, Boston, MA, USA). The age-total length relationship was modeled with an exponential function:

*y* = *ae^bx^*. (1)

where *x* represents age (dph) and *y* is total length. Allometric growth of organs was described by the power function:

*y* = *ax^b^*. (2)

where *x* denotes total length, *y* is organ length, *a* is the intercept, and *b* is the allometric exponent. Values of *b* = 1, *b* > 1 and *b* < 1 indicate isometric, positive allometric and negative allometric growth, respectively. When a clear change in growth rate was observed during development, a segmented regression approach was applied. Two separate power functions (*y* = *a*_1_*x^b^*^1^, *y* = *a*_2_*x^b^*^2^) were fitted to the data before and after a candidate inflection point using nonlinear least squares with the Levenberg–Marquardt iterative method. The optimal inflection point was determined by systematically testing candidate breakpoints across the range of total length and selecting the value that maximized the coefficient of determination (R^2^) and minimized the combined residual sum of squares of the two fits. A *t*-test assessed whether *b* differed significantly from 1. Prior to statistical analysis, normality (Shapiro–Wilk test) and homogeneity of variances (Levene’s test) were verified using the car package. If the assumptions were violated, the data were log10-transformed prior to ANOVA. Differences in enzyme activities among ages were analyzed by one-way ANOVA followed by Tukey’s HSD post hoc test using the dplyr package. The significance threshold was set at *p* < 0.05. All figures were prepared using Origin (version 2018C; OriginLab Corporation, Northampton, MA, USA).

## 3. Results

### 3.1. Allometric Growth Patterns of C. guichenoti

**Figure 2:**
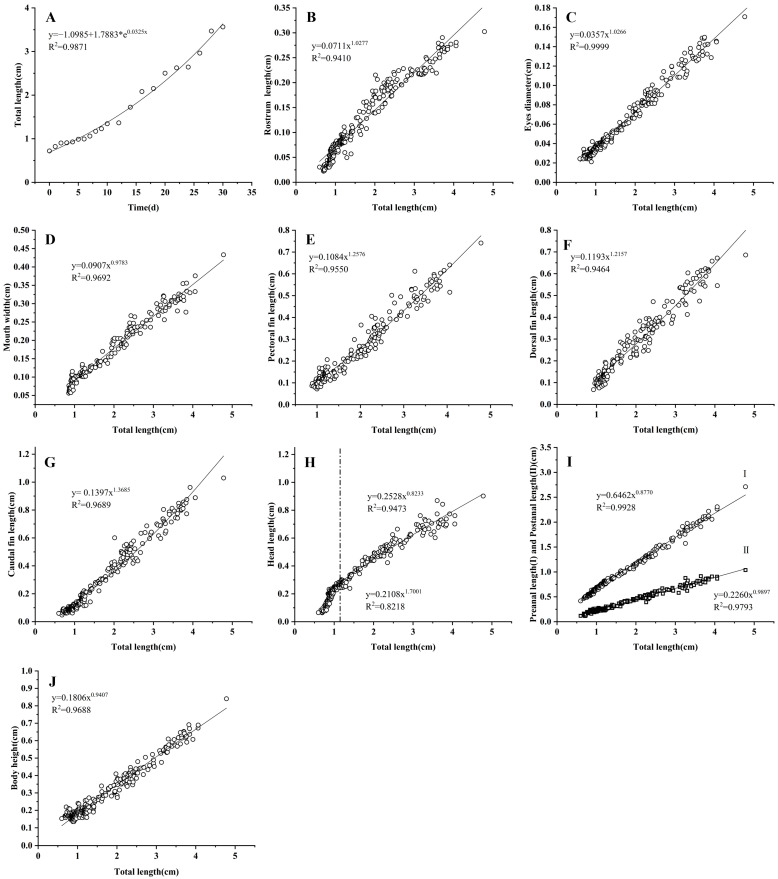
Correlation between total length and age (in days), allometric growth curve and function of organs and body parts in *C. guichenoti* from 0 to 30 days post-hatching (dph). (**A**) Total length vs. Age. (**B**) Rostrum length. (**C**) Eye diameter. (**D**) Mouth width. (**E**) Pectoral fin length. (**F**) Dorsal fin length. (**G**) Caudal fin length. (**H**) Head length (the dotted line indicates the inflection point at 1.2 cm total length). (**I**) Pre-anal and post-anal length. (**J**) Body depth.

The total length of *C. guichenoti* larvae increased exponentially with age (Figure 2A), described by the equation: *y* = −0.1985 + 1.7883 × e^0.0325*x*^. where *x* represents age in days post-hatching (dph). At hatching (0 dph), mean total length was 0.72 ± 0.05 cm. By 30 dph, larvae reached a mean total length of 3.47 ± 0.48 cm, reflecting a gradual increase in growth rate over time.

No significant allometric growth was observed in rostrum length (Figure 2B) or mouth width (Figure 2D). In contrast, eye diameter exhibited significant positive allometric growth (*p* < 0.05, *b* = 1.0266) with no inflection point, indicating sustained rapid growth relative to body length (Figure 2C). Newly hatched larvae (0 dph) possessed well-developed caudal fins and rudimentary pectoral fins. Distinct pectoral fins emerged by 4–5 dph, followed by dorsal fins at 6–7 dph. All three fins showed significant positive allometric growth (*p* < 0.05): pectoral fins (*b* = 1.2576; Figure 2E), dorsal fins (*b* = 1.2175; Figure 2F), and caudal fins (*b* = 1.3685; Figure 2G). These results indicate accelerated fin development throughout the early larval stage.

Head length of *C. guichenoti* exhibited a clear inflection point at approximately 1.2 cm total length (10–12 dph) (Figure 2H). From 0 to 12 dph, head length showed positive allometric growth, with an allometric exponent (*b* = 1.7001) significantly greater than 1 (*p* < 0.05). From 12 to 30 dph, the exponent decreased to *b* = 0.8233 (*p* < 0.05), indicating a shift to negative allometric growth.

Post-anal length displayed no significant allometric variation throughout development (Figure 2I). In contrast, pre-anal length exhibited significant negative allometry, with an exponent of b = 0.8770 (*p* < 0.05) (Figure 2I). Body depth also followed a negative allometric pattern (b = 0.9407, *p* < 0.05) during the study period (Figure 2J). The detailed growth coefficients and statistical parameters for these allometric relationships are provided in Appendix A Table A1.

### 3.2. Histological Observation of Digestive System Development in C. guichenoti

**Figure 3:**
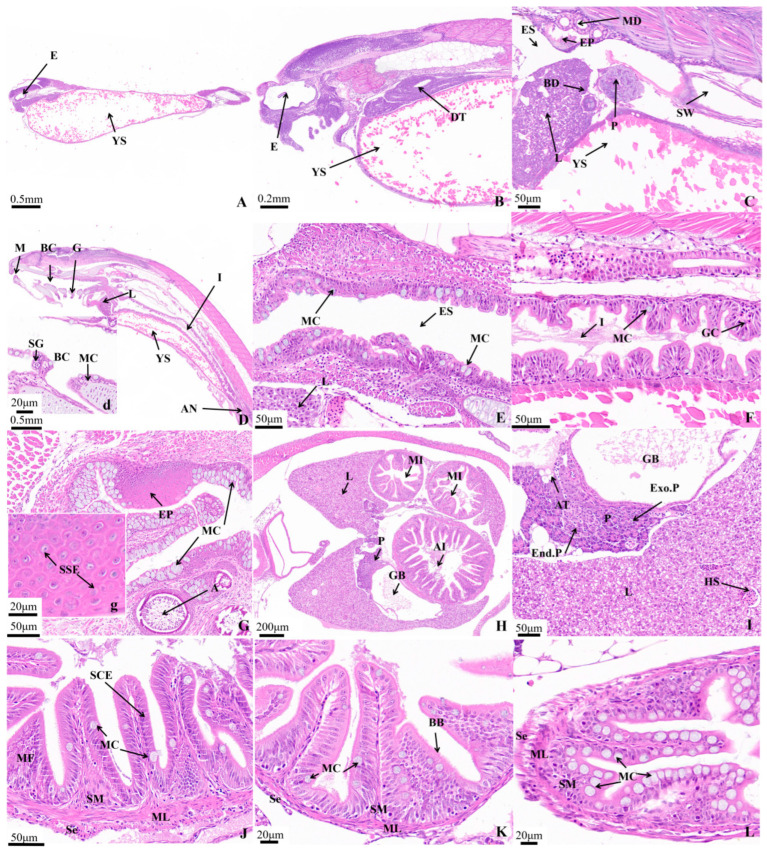
Histological observations of the digestive system in *C. guichenoti* from 0 to 5 days post-hatching (dph). (**A**) Longitudinal section of newly hatched larvae fish. (**B**) Longitudinal section of 1 dph larvae fish. (**C**) Longitudinal section of 2 dph larvae fish. (**D**,**d**) Longitudinal section of 4 dph larvae fish and buccopharyngeal cavity at 4 dph. (**E**) Longitudinal section of esophagus at 4 dph. (**F**) Longitudinal section of intestine at 4 dph. (**G**,**g**) Longitudinal section of esophagus of 5 dph larvae fish and esophageal papillae at 5 dph. (**H**) Transverse section of abdomen at 5 dph. (**I**) Longitudinal section of hepatopancreas at 5 dph. (**J**) Transverse section of anterior intestine at 5 dph. (**K**) Transverse section of middle intestine at 5 dph. (**L**) Transverse section of posterior intestine at 5 dph. A: Artery; AI: Anterior intestine; AN: anus; AT: Adipose tissue; BB: Brush border; BC: Buccopharyngeal cavity; BD: Bile duct; DT: Digestive tract; E: Eye; End.P: Endocrine pancreas; Exo.P: Exocrine pancreas; EP: Esophageal papilla; ES: Esophagus; G: Gill; GB: Gall bladder; GC: Goblet cell; HS: Hepatic sinusoid; I: Intestine; L: Liver; M: Mouth; MC: Mucous cell; MD: Mucous duct; MF: Mucosal fold; MI: Middle intestine; ML: Muscular layer; P: Pancreas; SCE: Simple columnar epithelium; Se: Serosa; SG: salivary gland; SM: Submucosa; SSE: stratified squamous epithelium; SW: Swimming bladder; YS: Yolk sac.

Newly hatched larvae (7.21 ± 0.32 mm TL) possessed a large yolk sac, and the digestive system was undeveloped with a non-patent oropharyngeal cavity (Figure 3A). By 1 dph, the oral cavity began to form, and an undifferentiated digestive tract appeared between the yolk sac and spine (Figure 3B). At 2 dph, the oropharyngeal cavity and esophagus were largely formed. Papillary structures appeared at the posterior esophagus, covered by squamous epithelial cells containing multiple mucus ducts. Concurrently, the liver and pancreas appeared as independent cell clusters (Figure 3C).

At 4 dph, the yolk volume was substantially reduced. The oropharyngeal cavity, esophagus, and intestine were clearly differentiated and patent to the anus. Salivary glands and mucous cells were present in the oropharyngeal region (Figure 3D), with mucous cells also appearing in the esophageal and intestinal mucosa (Figure 3E,F). By 5 dph, the yolk sac was fully absorbed. Esophageal papillae were enlarged and thickened (Figure 3G). The intestine elongated into an N-shaped loop (Figure 3H), with distinct wall layers (mucosa, submucosa, muscularis, serosa). Mucosal folds increased in complexity (Figure 3J). Regional differentiation was evident: the foregut displayed a thicker muscular layer; brush borders emerged in the foregut and midgut (Figure 3J,K); and mucous cell abundance increased in the hindgut (Figure 3L). The liver and pancreas expanded rapidly, with pancreatic tissue differentiating into endocrine islets and exocrine cells containing zymogen granules (Figure 3I).

**Figure 4:**
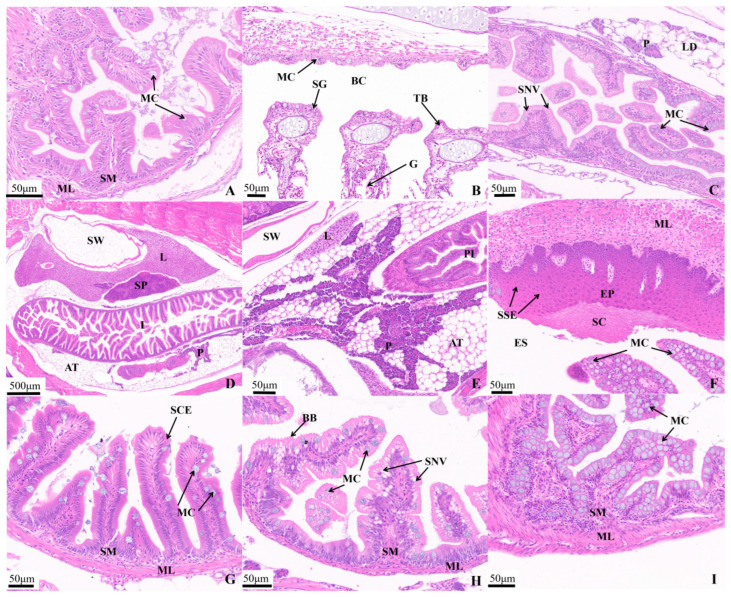
Histological observation of the digestive system in *C. guichenoti* from 10 to 30 days post-hatching (dph). (**A**) Transverse section of anterior intestine at 10 dph. (**B**) Longitudinal section of buccopharyngeal cavity at 15 dph. (**C**) Longitudinal section of posterior intestine at 20 dph. (**D**) Longitudinal section of abdomen at 20 dph. (**E**) Longitudinal section of hepatopancreas at 20 dph. (**F**) Longitudinal section of esophagus at 25 dph. (**G**) Transverse section of anterior intestine at 30 dph. (**H**) Transverse section of middle intestine at 30 dph. (**I**) Transverse section of posterior intestine at 30 dph. AT: Adipose tissue; BB: Brush border; BC: Buccopharyngeal cavity; EP: Esophageal papilla; ES: Esophagus; G: Gill; L: Liver; MC: Mucous cell; ML: Muscular layer; P: Pancreas; SC: Stratum corneum; SCE: Simple columnar epithelium; SG: salivary gland; SM: Submucosa; SNV: Supranuclear vacuole; SP: Spleen; SSE: stratified squamous epithelium; SW: Swimming bladder; TB: Taste bud.

At 10 dph, foregut mucosal complexity increased with secondary folding (Figure 4A). Taste buds were observed in the oropharyngeal cavity by 15 dph (Figure 4B). By 20 dph, the digestive system was fully developed with clear demarcation between midgut and hindgut (Figure 4C). Midgut epithelial cells contained numerous supranuclear vacuoles, indicating active absorption. The hindgut showed a thicker muscular layer and more mucous cells. The liver and pancreas began to fuse into a diffuse hepatopancreatic complex surrounded by adipose tissue (Figure 4D,E). At 25 dph, esophageal papillae increased in size, and surface squamous epithelial cells keratinized (Figure 4F). By 30 dph, the foregut, midgut, and hindgut showed further maturation in mucous cell proliferation, structural complexity, and muscular development (Figure 4G,I).

In summary, the yolk sac was depleted within 5 dph. The digestive tract became patent by 4 dph, and functional structures were present by 5 dph, marking the onset of exogenous feeding. Rapid differentiation occurred from 5 to 20 dph, with structural maturity largely achieved by 20 dph. A comprehensive summary of the major morpho-physiological events observed during the ontogeny of *C. guichenoti* is presented in Appendix A Table A2.

### 3.3. Changes in Digestive Enzyme and Alkaline Phosphatase Activities in C. guichenoti

**Figure 5:**
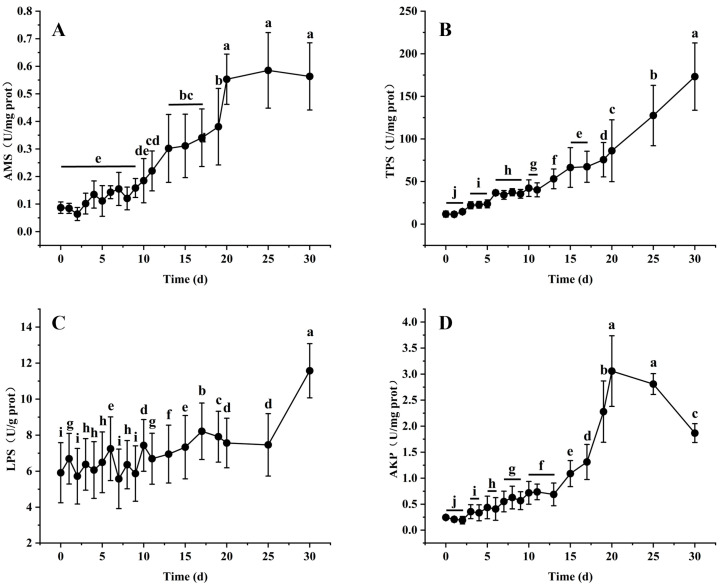
Activities of digestive enzymes in larval *C. guichenoti* from 0 to 30 days post-hatching (dph). (**A**): Amylase; (**B**): Lipase; (**C**): Trypsin; (**D**): Alkaline phosphatase. Different lowercase letters indicate significant differences among groups (*p* < 0.05).

Activities of amylase, lipase, trypsin, and alkaline phosphatase were detected in newly hatched larvae (Figure 5). Amylase activity increased progressively from 0 to 15 dph, rose sharply between 15 and 20 dph to reach a peak, and then stabilized from 20 to 30 dph (Figure 5A). Activity during the plateau phase (20–30 dph) was significantly higher than at 15 dph (*p* < 0.05).

Lipase activity remained relatively low compared to amylase and trypsin (Figure 5B). No significant changes were detected from 0 to 25 dph; however, a marked increase occurred at 30 dph (*p* < 0.05). Trypsin activity was the highest among all enzymes and increased consistently throughout development (Figure 5C). Activity at 15 dph was significantly elevated relative to hatching and continued to rise, peaking at 30 dph (*p* < 0.05). Alkaline phosphatase activity showed a unimodal pattern, increasing to a peak around 20 dph before declining slightly, with no significant difference between 25 and 30 dph (Figure 5D).

## 4. Discussion

The early developmental stage of fish is characterized by rapid morphological differentiation, where allometric growth patterns reflect adaptive responses to specific ecological pressures [2,4,11]. In this study, *C. guichenoti* larvae exhibited pronounced positive allometric growth (*b* > 1) in swimming organs (pectoral, dorsal, and caudal fins) and eye diameter, contrasting with the negative allometry observed in body depth. From a biomechanical and energetic perspective, this streamlined body form improves swimming coordination and reduces energy expenditure during prey capture [14,15,23]. This pattern aligns closely with findings in other rheophilic cyprinids of the Yangtze basin; for instance, He et al. [25,26] reported a similar prioritization of fin and sensory development in *Percocypris pingi pingi*, suggesting a convergent evolutionary strategy among riverine fishes to cope with high-velocity currents. This distinct trajectory likely represents a direct adaptation to the lotic habitat of the Jinsha River [14,15,23].

In swift currents, larvae must develop effective locomotor capabilities immediately after hatching to maintain position and avoid being swept downstream [27]. Specifically, the rapid development of fins enhances maneuverability for rheotaxis and supports balance control during prey capture [28,29]. Meanwhile, accelerated eye growth aids visual prey detection in turbid waters [30]. Furthermore, while Zeng and Liu [31] noted that pharyngeal adaptations for feeding are phylogenetically deep-rooted in the Gobioninae, our data show rapid allometric growth of fins and eyes alongside a later maturation of the digestive system, which is consistent with a developmental strategy that prioritizes locomotor and sensory capabilities over feeding structures during the larval stage. This “survival-first” strategy ensures larvae can navigate turbulent environments before engaging in adult benthic foraging [18].

Parallel to these morphological changes, the histological ontogeny of the digestive system reveals unique structural specializations adapted to a stomachless lifestyle. The absence of a stomach necessitates alternative mechanisms for food processing, and our observation of well-developed esophageal papillae covered by keratinized squamous epithelium is consistent with preliminary findings in the related species *Rhinogobio ventralis* [20]. These papillae likely serve as a compensatory mechanical grinder [9,10], pre-processing hard-shelled benthic invertebrates—which constitute the primary adult diet [10,18,32,33]—before they enter the intestine.

The timeline of maturation identified 5 dph as a critical milestone, marking the convergence of lumenization, hepatopancreas differentiation, and the onset of exogenous feeding capacity. Digestive gland development was closely associated with larval digestive and absorptive capacity [5,34]. Liver, pancreas, and gallbladder were discernible by 2 dph. By 5 dph, coinciding with complete yolk absorption, hepatic and pancreatic tissues had expanded, with hepatocytes showing vacuolation indicative of glycogen storage and early energy allocation, likely derived from repackaged yolk nutrients. This strategy may represent an evolutionary adaptation to pre-feeding energy deficits [35,36]. While Liu et al. [24] observed similar enzymatic and growth patterns in the common carp (*Cyprinus carpio*), the delayed full maturation of the midgut mucosa in *C. guichenoti* (until 20 dph) suggests a more cautious developmental pace, likely an adaptation to the colder, oligotrophic conditions of the upper Yangtze compared to warm-water pond species. The formation of a diffuse hepatopancreatic complex by 20 dph further indicates an advanced metabolic capacity for nutrient storage [7], a trait critical for surviving fluctuating food availability in river systems [37,38].

The temporal profiles of digestive enzymes provide further insight into the regulatory mechanisms governing this development, and are consistent with the “endogenous programming” hypothesis [34,39,40]. Trypsin activity was dominant throughout, peaking at 30 dph, which aligns with the high protein demand for somatic growth. This is consistent with the biochemical composition of the live feeds used in this study: rotifer *Keratella cochlearis* (crude protein: 59.4 ± 3.1%, crude lipid: 8.1 ± 1.3%), cladoceran *Bosmina longirostris* (crude protein: 57.8 ± 4.5% protein, crude lipid: 9.2 ± 2.0%), and copepod *Mesocyclops leuckarti* (crude protein: 60.3 ± 4.1%, crude lipid: 16.3 ± 1.6%). The protein-rich nature of these prey items (55–65% on a dry weight basis) suggests a relationship with the persistently high trypsin activity observed throughout the larval stage [18,32].

In contrast, amylase and alkaline phosphatase (AKP) exhibited unimodal patterns peaking at 20 dph. The surge in amylase coincides with the documented maturation of the hepatopancreas and may reflect preparation for the omnivorous adult diet [41], while the AKP peak coincides with increased microvilli density, which is consistent with the view that optimal absorption capacity lags behind structural formation as reported in previous studies [42,43]. Notably, AKP activity declined after 20 dph, which is consistent with observations in other cyprinids such as common carp (*Cyprinus carpio*) and silver pomfret (*Pampus argenteus*), where AKP decreases after intestinal structural maturation is complete [24,34]. This decline is consistent with a transition from active differentiation to a steady state of nutrient absorption.

Lipase activity remained relatively low throughout the first 25 dph, increasing only modestly by 30 dph. This pattern shows a temporal correlation with the lipid content of the live feeds. During early development (5–15 dph), larvae were fed primarily rotifers (6–12% lipid) and juvenile cladocerans (8–15% lipid). The lipid content of these prey compared to protein levels may reduce the functional demand for lipolytic capacity during the early larval stage. The slight increase in lipase activity at 30 dph coincides with the increased proportion of copepods (16.3 ± 1.6% lipid) in the diet after 15 dph, suggesting a potential responsiveness of lipase activity to dietary lipid levels [44,45,46]. However, direct experimental manipulation of dietary lipid content would be required to establish causality. It is also possible that early lipid deposition partially depends on endogenous synthesis from proteins and carbohydrates [41,42].

Integrating these morphological, histological, and physiological data reveals a sophisticated, staged adaptive strategy employed by *C. guichenoti* to navigate the high-mortality “critical period” [1]. During the initial endogenous phase (0–5 dph), our results are consistent with the hypothesis that limited yolk energy is strategically allocated to position holding and predator evasion rather than digestive capacity. In the swift Jinsha River, a larva that can feed but cannot maintain its position will drift to death; thus, locomotor competence appears to be the primary prerequisite for survival. This is followed by a critical transition phase around 5 dph, where sufficient locomotor control, structural gut readiness, and a surge in proteolytic activity converge. This precise timing minimizes the “mismatch” risk described in the Match-Mismatch Hypothesis [6], ensuring that when the larva needs to feed, it possesses both the mechanical ability to capture prey and the physiological capacity to digest it. As larvae transition to juveniles (15–25 dph), the focus shifts toward maximizing nutrient absorption and energy storage, optimizing the digestive system to support exponential growth. Only after this comprehensive maturation can the fish successfully adopt the benthic diet rich in mollusks and insects [19,20], a transition facilitated by the specialized pharyngeal structures evolving in this lineage [12].

The identification of critical developmental windows—5 dph for first feeding, 10–12 dph for the inflection point of cranial development, and 20 dph for digestive system maturity—provides a scientific basis for optimizing artificial rearing protocols for *C. guichenoti*. Specifically, live feeds with high protein content (such as the rotifers, cladocerans, and copepods used in this study) are recommended during the early larval stage (5–20 dph) to support the high trypsin activity and rapid growth. After 20 dph, when the digestive system is functionally mature, formulated diets with higher lipid content could be introduced, potentially coinciding with the observed increase in lipase activity.

## 5. Conclusions

This study systematically characterized the early ontogeny of allometric growth, digestive histology, and digestive enzyme activities in larval *C. guichenoti* from hatching to 30 dph. The results show positive allometric growth of swimming and sensory organs, consistent with a locomotion-first strategy for lotic survival. Histologically, the digestive tract becomes patent by 4 dph, functional structures are established by 5 dph coincident with the onset of exogenous feeding, and structural maturity is achieved by 20 dph. Digestive enzyme activities reveal distinct developmental profiles for protease, amylase, and lipase, reflecting the changing nutritional demands during larval development. Three critical windows are identified: 5 dph (initiation of exogenous feeding), 10–12 dph (cranial development inflection), and 20 dph (digestive system maturity). These windows provide a scientific basis for optimizing artificial rearing protocols, including the timing of first feeding, weaning strategies, and dietary transitions.

## Data Availability

The data presented in this study are available upon request from the corresponding authors.

## Appendix A

**Table A1:** Growth coefficients (a, b, R^2^) before and after inflection point.

Trait	Phase	*n*	*a*	*b*	Comparison (*b* vs. 1)	*p*-Value	R^2^
Rostrum length	0–30 dph	252	0.0711	1.0277	b = 1, *p* > 0.05	0.174	0.9410
Eye diameter	0–30 dph	252	0.0357	1.0266	b > 1, *p* < 0.05	<0.001	0.9999
Mouth width	0–30 dph	192	0.0907	0.9783	b = 1, *p* > 0.05	0.175	0.9692
Pectoral fin	0–30 dph	204	0.1084	1.2576	b > 1, *p* < 0.05	<0.001	0.9550
Dorsal fin	0–30 dph	180	0.1193	1.2175	b > 1, *p* < 0.05	<0.001	0.9464
Caudal fin	0–30 dph	252	0.1396	1.3685	b > 1, *p* < 0.05	<0.001	0.9689
Post-anal length	0–30 dph	252	0.2260	0.9897	b = 1, *p* > 0.05	0.303	0.9793
Pre-anal length	0–30 dph	252	0.6462	0.877	b < 1, *p* < 0.05	<0.001	0.9928
Body depth	0–30 dph	252	0.1806	0.9407	b < 1, *p* < 0.05	<0.001	0.9688
Head length	0–12 dph (TL < 1.2 cm)	144	0.2108	1.7001	b > 1, *p* < 0.05	<0.001	0.8218
Head length	12–30 dph (TL ≥ 1.2 cm)	108	0.2528	0.8233	b < 1, *p* < 0.05	<0.001	0.9473

**Table A2:** Major ontogenetic morpho-physiological changes.

Age (dph)	Developmental Events
0	Hatching; yolk sac present; caudal and pectoral fins rudimentary; digestive system undeveloped
2	Oropharyngeal cavity and esophagus formed; liver and pancreas appear
4	Digestive tract patent; salivary glands and mucous cells present
5	Yolk sac fully absorbed; exogenous feeding begins; intestinal looping and brush border appear
10–12	Inflection point for head growth (positive to negative allometry)
20	Digestive system structurally mature; hepatopancreas complex formed; peak amylase and AKP activities
30	Peak trypsin activity; lipase activity increases
